# Survey of strain distribution and antibiotic resistance pattern of group B streptococci (Streptococcus agalactiae) isolated from clinical specimens

**DOI:** 10.3205/dgkh000278

**Published:** 2016-09-12

**Authors:** Seyed Masoud Mousavi, Mona Nasaj, Seyed Mostafa Hosseini, Mohammad Reza Arabestani

**Affiliations:** 1 Department of Microbiology, Faculty of Medicine, Hamadan University of Medical Sciences, Hamadan, Iran; 2 Brucellosis Research Center, Hamadan University of Medical Sciences, Hamadan, Iran

**Keywords:** Streptococcus agalactiae, group B streptococci, capsular genotyping, polymerase chain reaction, antibiotic susceptibility, macrolide-lincosamide-streptogramin B

## Abstract

**Aim:** The aims of the present study were to determine the antibiotic susceptibility profils with particular emphasis on susceptible or resistant strains to macrolides and lincosamids antibiotics and to determine possible antibiotic resistance mechanisms occurring in group B streptococci (GBS) strains using PCR assay and disk diffusion method.

**Methods:** A total of 62 clinical GBS strains were investigated. Antibacterial susceptibility testing was performed using the disk diffusion method and inducible resistance test for clindamycin by standard double disk diffusion or D-zone test for all isolates to differentiate macrolide resistance phenotype (M), constitutive macrolide-lincosamide-streptogramin B phenotype (cMLS_B_) and induced macrolide-lincosamide-streptogramin B phenotype (iMLS_B_). In addition, minimum inhibitory concentrations (MIC) of penicillin were determined for all isolates. Finally, possible existence of antibiotic resistance genes for erythromycin (*ermTR*, *ermB* and *mefA/E*) and for clindamycin *(linB*) were examined among isolates using PCR assay.

**Results:** All 62 isolates were susceptible to penicillin, ampicillin, linezolid, cefazoline and vancomycin. However, 93.5% (n=58) of isolates showed an increased MIC to penicillin. The overall rate of erythromycin resistance was 35.5% (n=22). All erythromycin-resistant isolates displayed the M phenotype (100%, n=22). All three erythromycin resistance genes (i.e. *ermTR*, *ermB* and *mefA/E*) were found in erythromycin-resistant isolates.

**Conclusion:** It was concluded that prescribing antibiotic without antibacterial susceptibility tests should be prevented because of the high prevalence of erythromycin-resistant GBS strains and the fact that erythromycin-resistant GBS strains has shown an increased MIC to penicillin, as the drug of choice for treating GBS infections.

## Zusammenfassung

**Zielsetzung:** In der vorliegenden Studie wurden die Antibiotikaempfindlichkeit mit dem Schwerpunkt Empfindlichkeit gegen Makrolide und Linkosamide bestimmt und mögliche Resistenzmechanismen von Gruppe-B-Streptokokken (GBS) mittels PCR und Plättchendiffusionsmethode untersucht.

**Methode:** Es wurden 62 klinische GBS-Isolate untersucht. Die Resistenztestung wurde mit der Plättchendiffusionsmethode und der induzierbaren Resistenz gegen Clindamycin mittels Doppelplättchen-Diffusion oder D-Zonen-Test durchgeführt, um zwischen der phänotypischen Makrolidresistenz (M), der konstitutiven phänotypischen Makrolid-Linkosamid-Streptogramin B (cMLS_B_)- und der induzierbaren phänotypischen Makrolid-Linkosamid-Streptogramin B (iMLS_B_)-Resistenz zu differenzieren. Ergänzend wurde ebenfalls für alle Isolate die minimale Hemmkonzentration (MHK) für Penizillin bestimmt. Schließlich wurde die mögliche Existenz der Erythromycin- (*ermTR*, *ermB* und *mefA/E*) und Clindamycin-Resistenzgene (*linB*) mittels PCR untersucht.

**Ergebnisse:** Alle 62 Isolate waren gegen Penicillin, Ampicillin, Linezolid, Cefazolin und Vancomycin empfindlich. Allerdings zeigten 93,5% (n=58) der Isolate eine erhöhte MHK gegenüber Penicillin. Der Anteil Erythromycin-resistenter Isolate betrug 35,5% (n=22). Alle Erythromycin-resistenten Isolate repräsentierten den M-Phänotyp. In Erythromycin-resistenten Isolaten waren alle 3 Erythromycin-Resistenzgene (*ermTR*, *ermB* und *mefA/E*) nachweisbar.

**Schlussfolgerung:** Wegen der hohen Prävalenz Erythromycin-resistenter GBS-Stämme und der Tatsache, dass Erythromycin-resistente GBS-Stämme eine erhöhte MHK gegen Penicillin aufwiesen, sollten Infektionen durch GBS nicht ohne vorherige Resistenztestung behandelt werden.

## Introduction

Group B streptococci (GBS) are the main cause of life threatening infections in adults with diabetes or immune suppression and infants with sepsis or meningitis. Fatality rate related to GBS infections among infants has been estimated between 2% and 4%, however, this rate is even higher among premature infants [1], [2]. Penicillin and ampicillin are the first two choices in treating GBS infections and there is no report so far on GBS resistance to these antibiotics. The main problem here is that some people are allergic to penicillin and, as a result, we should attempt to develop alternative antibiotics for them. Macrolides (e.g. erythromycin) and lincozamides (e.g. clindamycin) are some useful options in this situation [3]. However, there is a significant volume of evidence suggesting that GBS are becoming resistant to erythromycin and clindamycin in both common population and pregnant women. According to statistics published by the Center of Disease Control (CDC), in vitro resistance level of GBS against both erythromycin and clindamycin increased between 25–32% and 13–20% during 2006–2009 [4]. GBS resistance to both macrolides and lincozamides occurs by methylation of the target region of 23s ribosomal in the large 50 S ribosomal subunit. Methylase, which causes the methylation of the target area, is encoded by erythromycin ribosomal methylase (*erm*) gene. More than 30 different *erm* genes have been found in various bacterial species. In *Streptococcus agalactiae*, *erm* (A) (subclass of *erm* (TR)) and *erm* (B) genes have been identified responsible for high-level resistance to erythromycin and clindamycin. In fact, the *Erm* enzyme, which induces cross-resistance to macrolides, lincosamides, and streptogramin B (MLS_B_), is known as the MLS_B_ phenotype, which classifies into two categories, the first one is the induced MLS_B_ resistance phenotype (iMLS_B_), and the second is the permanent MLS_B_ resistance phenotype (cMLS_B_) [4]. Another common mechanism of macrolide-induced resistance is an active efflux pump system, which is dependent on proton that drives out macrolides ring members 14 and 15 such as erythromycin from the bacterial cell membranes. This macrolide-specific efflux is encoded by *mef* genes; it is worth noting that *mef* (A) and *mef* (E) are identified in GBS. GBS isolates that carry *mef* are resistant to macrolides but they are vulnerable to lincozamides and streptogramines too [5]. *linB* is another antibiotic-resistant gene that encodes nucleotidyltransferase and donates phenotype L that is resistant to lincozamides and moderately sensitive to erythromycines. 

Because of the presence of antibiotic-resistant genes such as *ermB*, *ermTR*, and *ermA/E* on transposons, these genes can travel from an organism to another [6]. Accordingly, investigation and surveillance of GBS antibiotic resistance should be conducted regularly. According to the importance of the problem and aforementioned issues, the present study was carried out to achieve the following objectives; (1) determining the susceptibility of bacteria to antibiotics with a special focus on sensitive strains resistant to macrolides and lincozamides, (2) mechanisms of antibiotic resistance in bacteria in those cases that have shown resistance in the disk diffusion and PCR method. The study was carried out in Hamadan, Iran.

In fact, the present study was conducted to gain some knowledge about the susceptibility pattern of the GBS isolates in the area of the study. 

## Material and methods

### Sample collection, extraction, and detection

The study was performed from June 2013 to February 2014. A total of 62 clinical GBS isolates was collected, including 15 vaginal samples selected from 203 samples taken from pregnant women, one positive sample from a none pregnant women, 45 positive urine culture samples, and one positive blood culture sample. Moreover, 55 samples were collected from females and other six samples were obtained from males (all samples collected from males were UC). After conventional phenotypic identification tests, all these samples were inoculated in Brain Heart Infusion (BHI) broth medium containing 5% blood and 18% glycerol at the temperature of –70°C. GBS identification criteria using diagnostic phenotypic tests were as follows; beta-hemolytic colonies, gram-positive, chain-forming coccus, catalase positive, a positive result for the sodium hippurate hydrolysis test, a negative result for bile esculin agar test, antibiotic resistance to bacitracin disk and SXT, and a positive CAMP reaction. 

### DNA extraction and confirming GBS isolates using the PCR method

DNA was extracted by thermal lysis [7] (boiling method) with some modifications as follows: To concentrate the nucleic acid obtained from the boiling method, 400 µL of ice-cold ethanol was added into the micro tube containing nucleic acid, it was mixed gently and left in a deep freezer (–18 to –20°C) for 10–30 minutes; then, it was centrifuged for 10 minutes at the maximum speed. The supernatant was poured and the pellet was dried out thoroughly using the heater block (Nedaye Fan Co., Iran) at 55°C. In the final step, 50 µL of sterile 1x TE [Tris-EDTA buffer] buffer was poured to the micro tube and the pellet was gently dissolved. Some of the concentrated nucleic acid was utilized for PCR and the remaining was kept frozen at –20°C for following uses. 

In order to confirm the extracted isolates that were detected by phenotypic methods, PCR assay with an internal positive control of *atr* target gene, as a housekeeping gene, was applied and the reaction was done using Bio-Rad Thermal Cycler (the sequence of primer presented in Table 1 (Tab. 1)). It should be noted that for each PCR test, DNA samples of clinical isolates were simultaneously proliferated and evaluated with a positive control sample (DNA sample of *S. agalactiae*, ATCC 12386) and a negative control sample (DNA sample of *Enterococcus faecalis*, ATCC 29212).

### Antibiotics susceptibility 

The antibiotic susceptibility test for all samples was conducted using the following antibiotics; penicillin (10 units), ampicillin (10 µg), vancomycin (30 µg), tetracycline (30 µg), erythromycin (15 µg), azithromycin (15 µg), clindamycin (2 µg), cefazolin (30 µg), quinopriston/dalfopristin (synercid) (15 µg), chloramphenicol (30 µg), linezolid (30 µg), levofloxacin (5 µg) and doxycycline (30 µg). All antibiotics were manufactured by MAST Group Ltd. Concurrent with this test, the inducible resistance to clindamycin for all GBS isolates was determined using standardized double-disk (DD) diffusion test or D-zone test. In regard with D-zone test, the appearance of a D-shape halo on the medium was considered indicative of the inducible resistance to clindamycin, and accordingly, it was deduced whether there was a resistance to clindamycin or not. In the absence of such growth zones, the size of the non-growth zone for each antibiotic was measured and recorded. The interpretation of the susceptibility, resistance, or moderate resistance to antibiotics for each disk was performed based on the guidelines disseminated by CLSI 2014 [8]. 

In the cases of clindamycin and erythromycin, the regions lower than 15 mm around both disks is indicative of cMLS_B_ resistance. Furthermore, coincidence of resistance to erythromycin and lack of resistance to clindamycin is indicative of M phenotype. Finally, the coincidence of resistance to clindamycin and moderate resistance to erythromycin is indicative of L phenotype [4].

### MIC determination of the antibiotic penicillin

The lowest concentration of antibiotic penicillin able to inhibit the isolates was determined using gridlines recommended by CLSI 2014 [8].

### Tracking the presence of genes resistant to erythromycin and clindamycin using PCR method

In order to determine the specific resistance mechanisms, the PCR tests were conducted for all samples. For this purpose, *ermB*, *ermTR*, and *mefA/E* primers (by targeting genes resistant to erythromycin) for multiplex PCR and *linB* primer was used separately with the primer sequences presented in Table 1 (Tab. 1). 

### Data statistical analysis

The independent t-test was employed to compare the erythromycin antibiotic susceptibility and antibiotic susceptibility pattern of other antibiotics. The level of significance of the test was set at 0.05. All statistical analysis was performed using SPSS software package version 20.

## Results

The results of PCR with specific primers demonstrated that all 62 isolates were GBS strains. The results of the antibiotic susceptibility test using the disk diffusion method for erythromycin, clindamycin, and quinupriston/dalfopristin alongside these results for other antibiotics and the results of the broth microdilution method (MIC) for penicillin antibiotic are presented in Table 2 (Tab. 2).

The disk diffusion method was applied in order to determine the susceptibility pattern of erythromycin and clindamycin antibiotics; finally it was found that 22 strains (35.5%) were phenotype M, and also, there were not found iMLS_B_, cMLS_B_, and L phenotype in any strain. Furthermore, the results of multiplex PCR assays revealed that *ermTR*, *ermB*, and *mef A/E* genes were presented in 16.1, 19.4, and 12.9% of strains, respectively. The gene inducing resistance to clindamycin, *linB*, was identified in none of the strains. Simultaneous existence of genes resistant to erythromycin in GBS isolates are presented in Table 3 (Tab. 3), Table 4 (Tab. 4), Table 5 (Tab. 5), Table 6 (Tab. 6).

## Discussion

In the present study, all 62 clinical isolates that were examined using the disk diffusion method were susceptible to the antibiotics penicillin, ampicillin, linezolid, cefazolin and vancomycin. However, most of them, 58 cases (93.5%), had the increased minimum inhibitory concentration for penicillin. 

Furthermore, five isolates (8.1%) had the non-growth zone less than 17 mm (defined as the susceptibility limit criteria) for vancomycin disk. The overall frequency of resistance to erythromycin was 35.5% (22 cases). All isolates resistant to erythromycin were displayed M phenotype. All three genes inducing the resistance to erythromycin antibiotics *ermB*, *ermTR*, and *mefA/E*, were found in all isolates, which were resistant to this type of antibiotic. The presence of these genes was significantly related to the erythromycin susceptibility pattern (p<0.05) (Table 3 (Tab. 3) and Table 4 (Tab. 4)). However, one isolate resistant to erythromycin was identified that contained the *mefA/E* gene, which may be associated with the lack of expression of this gene in the isolate. The *ermB*, *ermTR*, and *mefA/E* genes were identified in 10 (16.1%), 12 (19.4%), and 8 (12.9%) cases, respectively. There were not observed any *linB* gene, which induce the resistance to clindamycin antibiotics. The presence of these three genes was not identified in isolates. In contrast, the simultaneous presence of the *ermTR* and the *ermB* genes was identified in three isolates, while only one isolate contained both *ermB* and the *mefA/E* genes simultaneously. 

The resistance to azithromycin, chloramphenicol, and levofloxacin was observed in 26, 2, and 3 cases, respectively, also all isolates were resistant to doxycycline, and tetracycline. It is interesting to note that none of the isolates were resistant to antibiotics clindamycin and quinupristin/dalfopristin. In this regard, current concerns are the resistance to macrolides and lincozamides that have been globally increased among GBS strains over two past decades; the frequency of resistance to erythromycin and clindamycin used to be less than 5%, but in recent years there have been reports explaining that these frequencies have been increased up to 54% in the United States. In Belgium, the prevalence of resistance to macrolide antibiotics among the invasive isolates increased from 10.4% in 2000 to 33% in 2008 [9]. These findings are in accordance with the reports from Europe [10], North America [11], and Asia [12], but some reports from Sweden explained these resistances lower than 10% [13]. The highest prevalence of resistance to erythromycin and clindamycin was reported by the study conducted by Dipersio et al.; in that study 200 GBS isolates were collected from vaginal and rectal specimens, from them, 54% were resistant to erythromycin and 33% were resistant to clindamycin [1]. In the study carried out by Florindo et al. in Portugal, the level of resistance to clindamycin and erythromycin was 10% and 19%, respectively. Moreover, in that study, all isolates were susceptible to penicillin and vancomycin, from 19 isolates resistant to erythromycin; 10 cases (53%) were cMLS_B_ phenotype, 8 cases (42%) were iMLS_B_ phenotype, and one case was M phenotype. The ermmethylase genes are related to the presence of mefA gene and were only found in M and MLS_B_ phenotype [14].

The results of other similar studies carried out in Iran are as follows; 115 isolates were analyzed by Emaneini et al., they reported that all the cases were susceptible to penicillin and quinupriston/dalfopristin antibiotics, but the level of their susceptibility to clindamycin, chloramphenicol, erythromycin, linezolid, and tetracycline were 35, 45, 35, 1, and 96%, respectively. Besides, in that study, the *ermTR* and *ermB* genes were identified in 13 and 16% of isolates, but there were no *ermA* and *mefA* in the isolates. All isolates resistant to erythromycin were cMLS_B_ phenotype, 22 genes contained only the *ermTR* gene and 5 isolates contained both *ermB* and *ermTR* genes [15]. In another study conducted by Janati et al. in Ardabil, Iran, the MIC method was used for determining the pattern of antibiotic susceptibility and the following results were reported; from 56 GBS isolates, there was no case resistant to erythromycin and only three cases were moderately resistant to this antibiotic. In the case of clindamycin, two isolates (3.5%) had a moderate resistance and two isolates were totally resistant. All isolates were susceptible to ampicillin, vancomycin, and penicillin and one isolate (1.7%) exhibited the reduced susceptibility pattern to penicillin [16]. 

The difference between the results gained by that study and those obtained in the present study can be attributed to the geographical differences between the regions the studies have been done. According to the CLSI guidelines, the presence of the beta-hemolytic Streptococcus isolates resistant to betalactam antibiotics have not been identified yet [8]. However, in the present study, the lack of the susceptibility of 58 clinical isolates was demonstrated using MIC method that is in accordance with other studies conducted in this area [17], [18], [19], [20].

In this study, the results of MIC examinations indicated that these 58 isolates had the MIC non-susceptible to penicillin [8]. The incidence of GBS isolates with reduced susceptibility to penicillin antibiotics in different regions of the world emphasizes on the need for establishing a continuous surveillance system on the increased MIC to penicillin antibiotics among GBS isolates [21]. The M phenotype with the frequency of 35.5% was the most prevalent one among isolates resistant to erythromycin. The prevalence of the M phenotype in other countries is as follows: Canada, 15%; France, 6–7.4%; Spain, 5–9.3%; and Taiwan, 37% [22]. In the present study, all three genes inducing resistance to erythromycin contributed in developing M phenotype (p<0.05) (Table 5 (Tab. 5)). This founding is in contradiction with the results of several studies which reported that the *mefA/E* gene was the only contributor in developing M phenotype, while these results are compatible with the results obtained by Seo et al. who reported that *mefA*, *mefB*, and *ermA* were associated with M phenotype [23]. 

Furthermore, in the present study, there was not found any significant association between the erythromycin antibiotic susceptibility pattern and the susceptibility pattern of other antibiotics other than azithromycin, because of the similarity of families, (p>0.05). Two isolates were concurrently resistant to chloramphenicol and erythromycin, but a significant association between resistance patterns of these two antibiotics was not observed. Likewise, two isolates of three ones that were resistant to levofloxacin were concurrently resistant to erythromycin, but there was no significant association between them (p=0.067) (Table 6 (Tab. 6)).

## Conclusion

In this study, it was found that a high proportion of the isolates had an increased MIC to penicillin. The incidence of GBS isolates with a reduced MIC to penicillin suggests that there is a possibility of future failure in treating the infections caused by these bacteria, especially in the case of consuming these antibiotics before or during delivery. According to the results obtained in this study, the antibiotics clindamycin, quinupriston/dalfopristin, linezolid, levofloxacin, vancomycin, and, chloramphenicol can be reliable alternatives of erythromycin in treating or preventing GBS infection in women who suffer from colonization or patients that are allergic to penicillin in Hamadan.

## Notes

### Competing interests

The authors declare that they have no competing interests.

## Figures and Tables

**Table 1:**
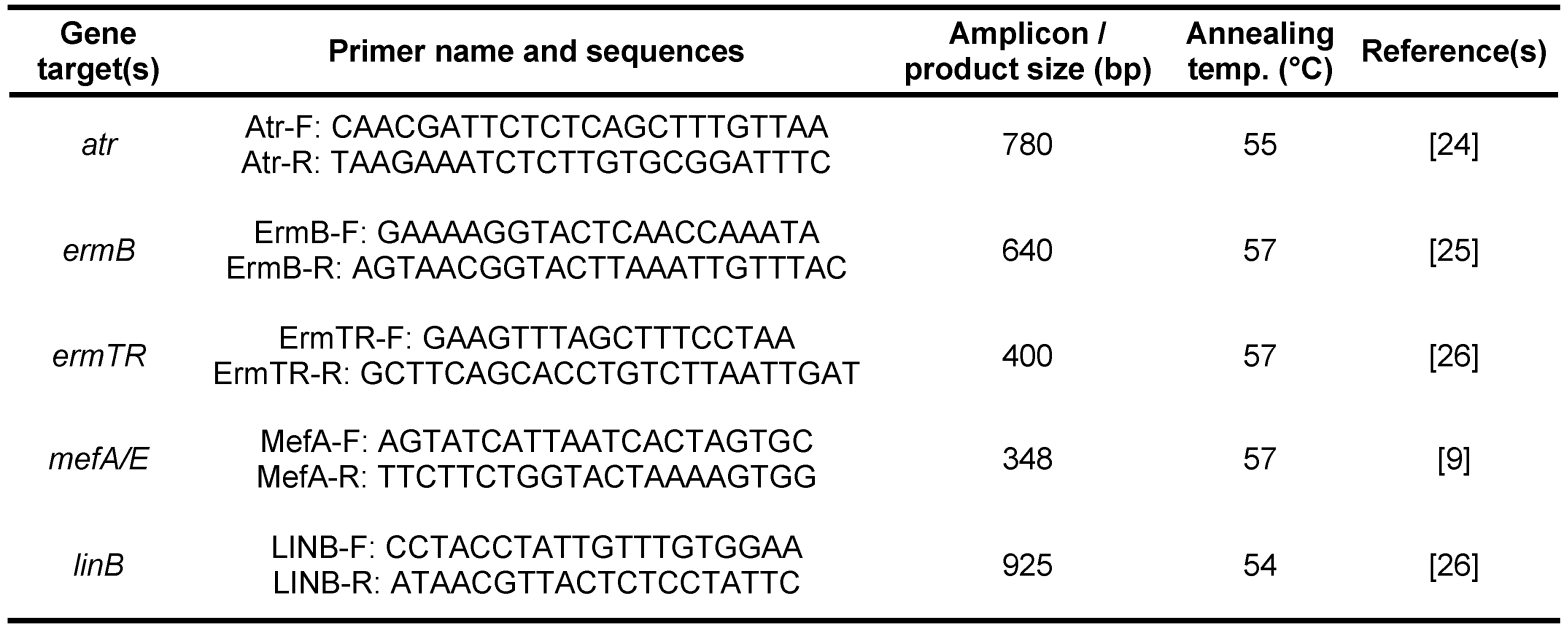
Table1: Primers used for PCR assay in this study

**Table 2:**
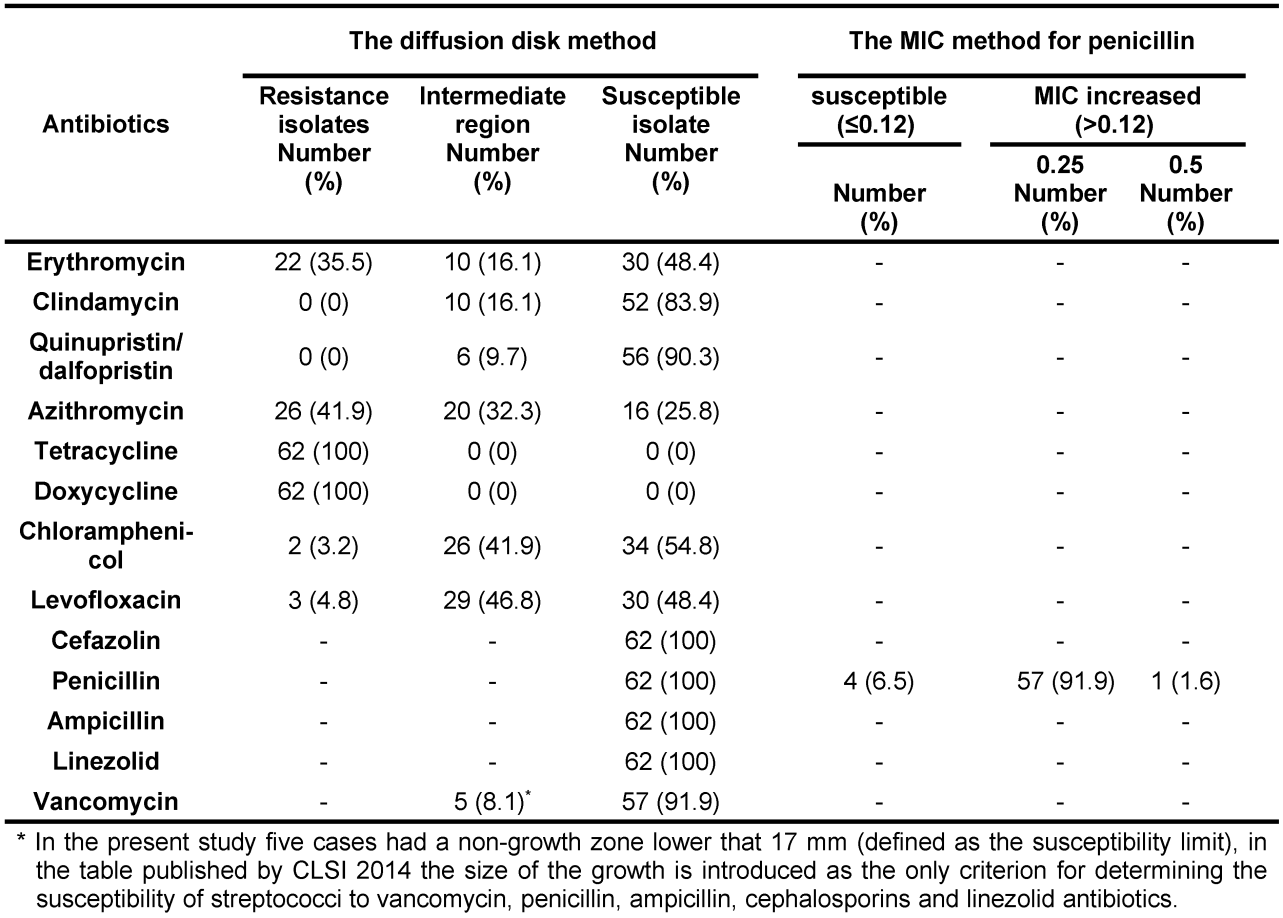
The results of antibiotics susceptibility test

**Table 3:**
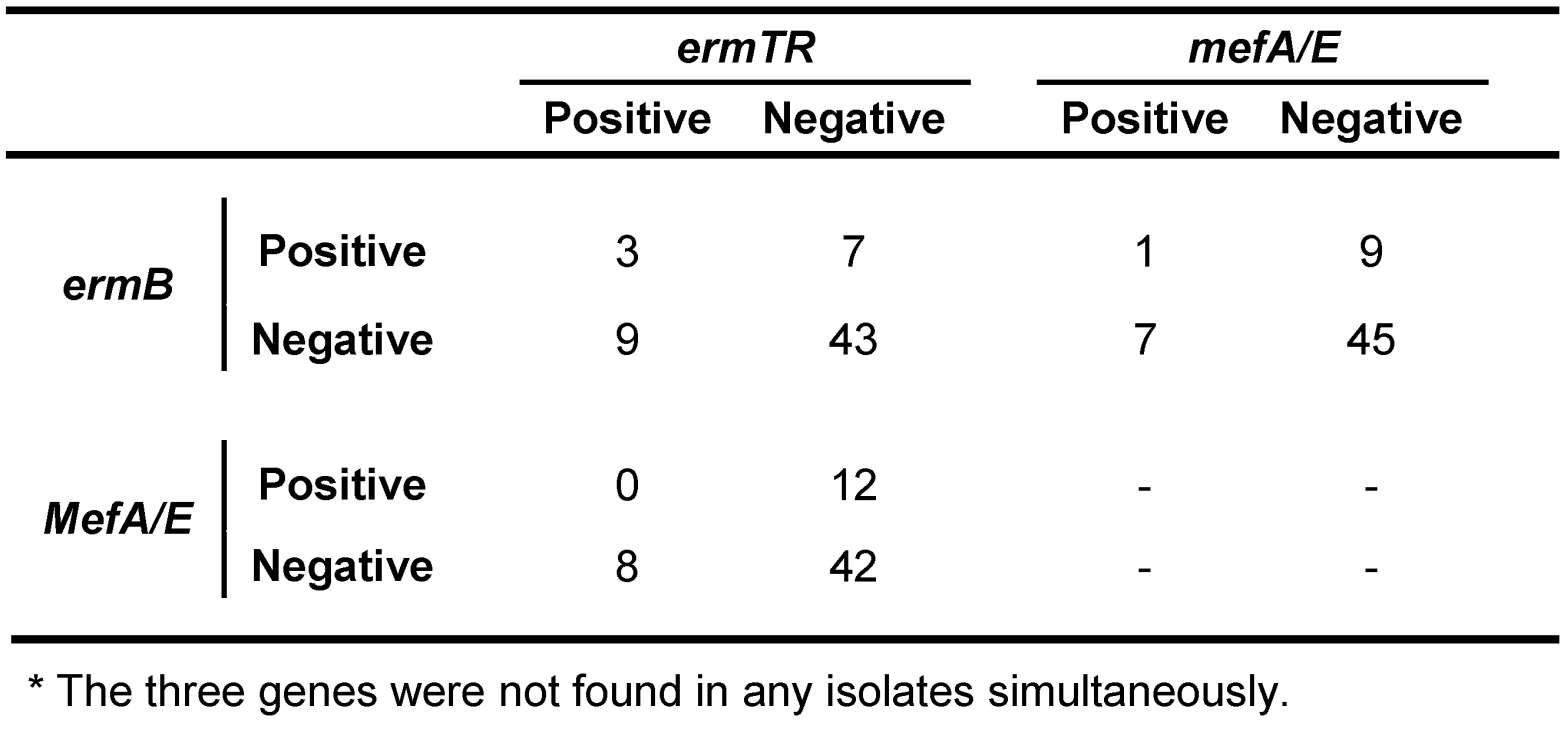
Comparison of the PCR assay for *mefA/E*, *ermTR*, and ermB*

**Table 4:**
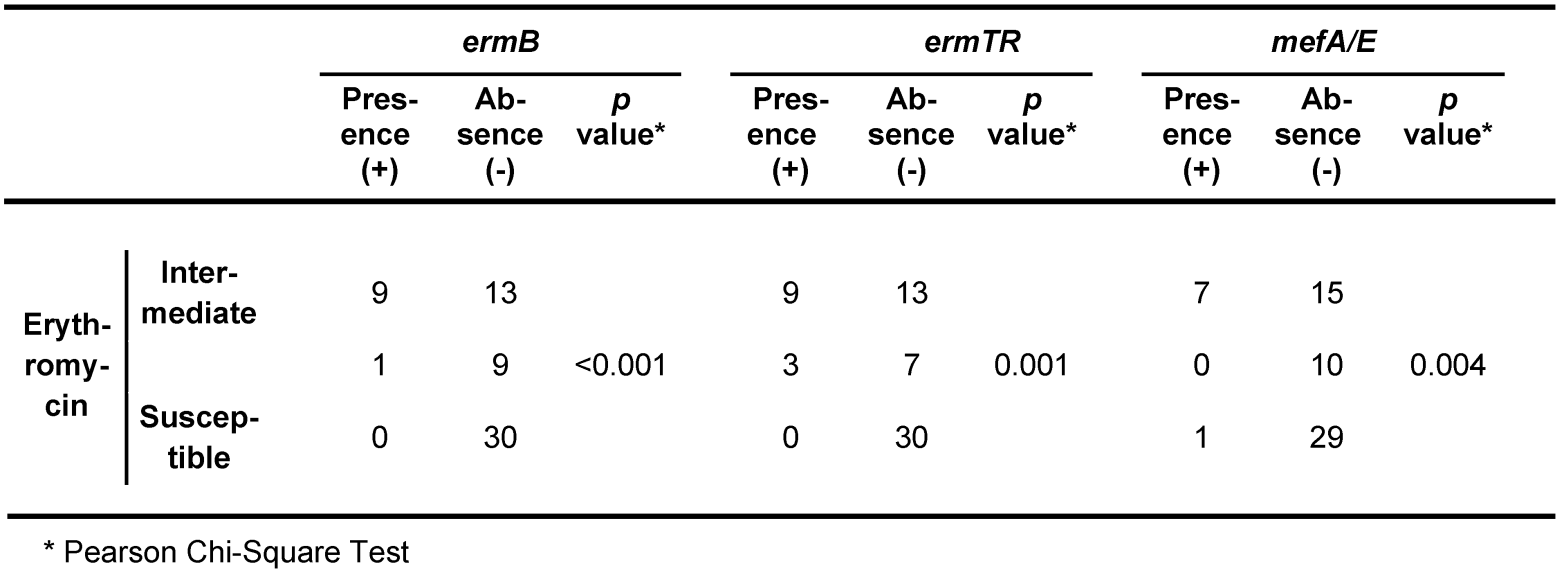
The association among genes resistant to antibiotics and the susceptibility pattern of erythromycin antibiotic

**Table 5:**
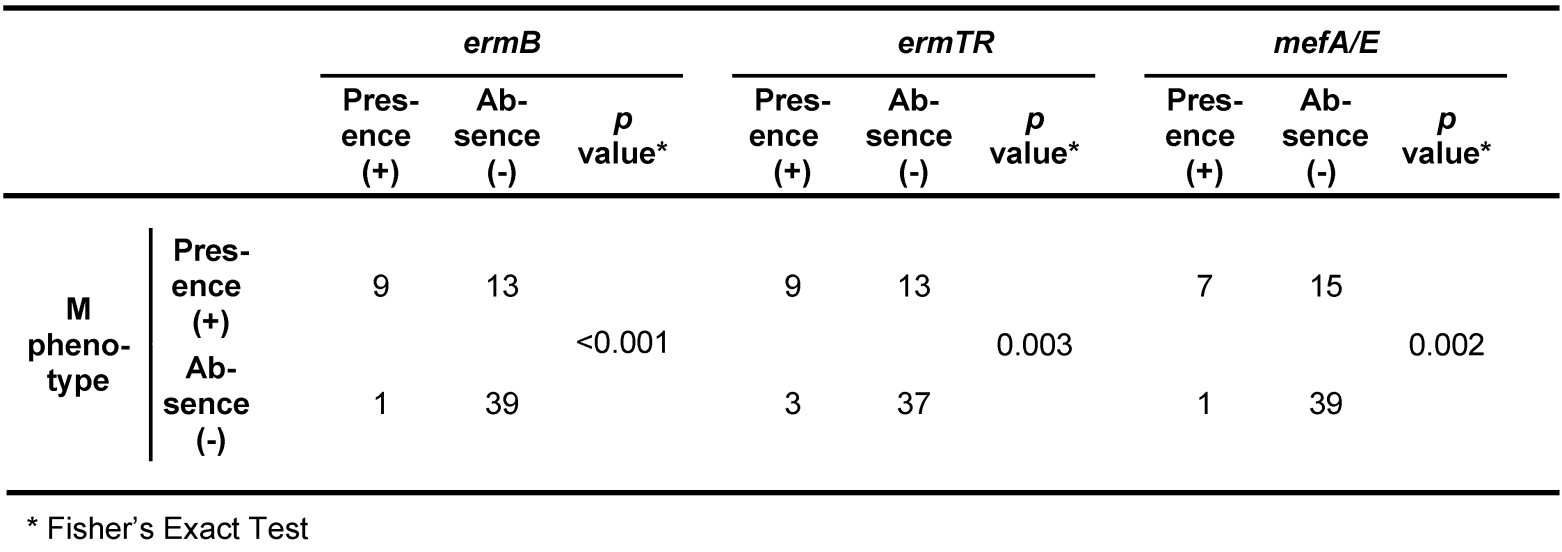
The association between resistant genotypes and phenotype M

**Table 6:**
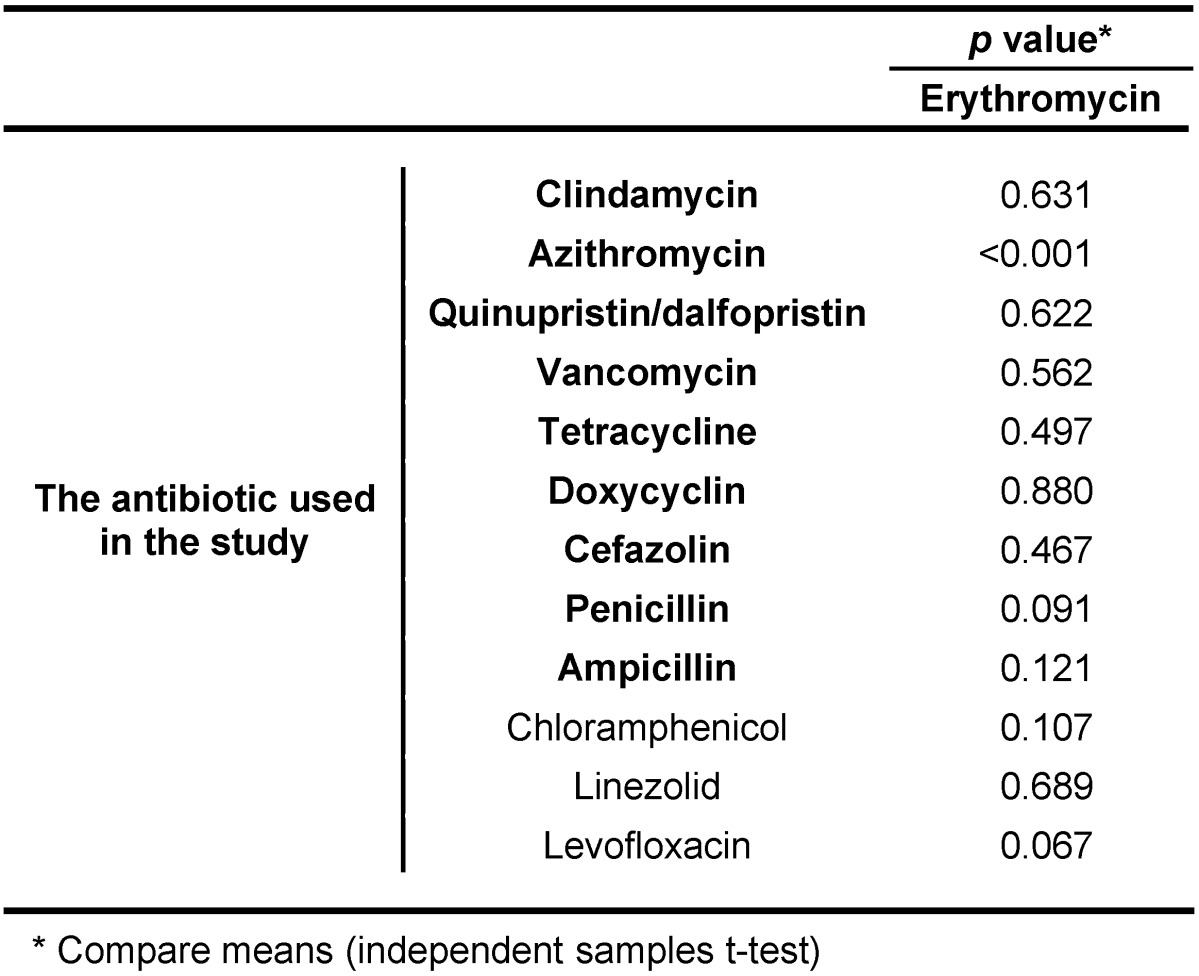
The association between the susceptibility patterns of erythromycin antibiotic and the susceptibility patterns of other antibiotics
